# A common polymorphic allele of the LH beta-subunit gene is associated with higher exogenous FSH consumption during controlled ovarian stimulation for assisted reproductive technology

**DOI:** 10.1186/1477-7827-11-51

**Published:** 2013-06-01

**Authors:** Carlo Alviggi, Kim Pettersson, Salvatore Longobardi, Claus Yding Andersen, Alessandro Conforti, Pasquale De Rosa, Roberto Clarizia, Ida Strina, Antonio Mollo, Giuseppe De Placido, Peter Humaidan

**Affiliations:** 1 Dipartimento Universitario di Scienze Ostetriche Ginecologiche e Medicina della Riproduzione, Area Funzionale di Medicina della Riproduzione ed Endoscopia Ginecologica, Università degli Studi di Napoli ‘Federico II’, Naples, Italy; 2 Department of Biotechnology, University of Turku, 20520, Turku, Finland; 3 Department of Medical Affairs, Merck-Serono Italia (S.L.), Rome, Italy; 4 Laboratory of Reproductive Biology, University Hospital of Copenhagen, Faculty of Health Science, University of Copenhagen, Copenhagen, Denmark; 5 The Fertility Clinic, Odense University Hospital (OUH), Odense, Denmark

**Keywords:** LH, Beta-subunit variant, Immunoassays, Polymorphism, Pharmacogenomics, IVF, Poor responders

## Abstract

**Background:**

V-betaLH is a common genetic variant of LH caused by two polymorphic base changes in the beta subunit gene, altering the amino acid sequence (Trp8Arg and Ile15Thr). In a previous-preliminary trial performed in women undergoing IVF, it was demonstrated that carriers of v-betaLH show sub-optimal ovarian response to a standard long GnRH-agonist down -regulation protocol when stimulated with pure recombinant FSH (r-hFSH). The aim of this study was to confirm the hypothesis that women with v-betaLH display hypo-sensitivity to exogenous FSH in a larger IVF population and to explore the frequency of this variant in a Danish female population.

**Methods:**

In the present study, the effect of v-betaLH was retrospectively investigated in a larger series of women undergoing controlled ovarian stimulation (COS) and, for the first time, in a Danish IVF population. A total of 220 normogonadotrophic women following a long GnRH-agonist down-regulation protocol received an individualized dose of r-hFSH (100 IU and 375 IU s.c. daily) according to antral follicle count, baseline FSH, body mass index and age. The LH genotype was assessed in all patients by immunofluorometric assay.

**Results:**

V-betaLH was present in 11% of patients, whereas the allelic frequency was 12%. The study population was divided into two groups according to their LH genotype. Group A consisted of 196 wt/wt women. Group B included 24 individuals with v-betaLH (21 heterozygous and 3 homozygous). No statistically significant differences in the mean number of oocytes retrieved, fertilization rate and pregnancy rate per cycle were observed between groups. However, Group B received a significantly higher cumulative-dose of r-hFSH than Group A (2435.86 +/− 932.8 IU versus 1959.8 +/− 736.45 *p* = 0.048). When one-way ANOVA in a within design was applied, the LH genotype had a statistically significant effect (*p* < 0.01) on the cumulative dose of r-hFSH, showing a progressive increase from wt/wt (1959.8 +/− 736.45 IU) to v-betaLH hetero- (2267.5 +/− 824.3) and homozygotic women (3558.3 +/− 970.9).

**Conclusions:**

These results confirm that carriers exhibit hypo-sensitivity to exogenous FSH during COS, documenting that the frequency of v-betaLH in Denmark is similar to a number of European countries.

## Background

More than 80% of patients receiving stimulation with exogenous *follicle-stimulating hormone* (FSH), devoid of any *luteinizing hormone* (LH) activity during IVF/ICSI, respond adequately to the stimulation in terms of follicular development and steroid synthesis. However, approximately 10% of patients require a higher dose of recombinant human FSH (r-hFSH) to obtain an optimal response. This subgroup of patients was recently classified as *hypo-responder patients*[1-5]. In contrast to the classical *poor-responder*, this type of patient possesses a normal ovarian reserve and follicular recruitment, however, requires more r-hFSH than a *normal-responder* patient during controlled ovarian stimulation (COS).

Although the attenuated response of *the hypo-responder* patient has not yet been identified, recent data suggest a possible role of LH. Firstly, it was demonstrated that supplementation with exogenous LH could be a useful strategy to improve ovarian response in this type of patient [2-4]. Thus, the use of recombinant human LH (r-hLH) supplementation during stimulation normalized the ovarian response and decreased the r-hFSH consumption [6]. Interestingly, the base line endogenous LH levels in these patients were similar to normal responding women, and it was postulated that different bioactive form of LH, known as v-betaLH, could characterize this type of patient; a hypothesis which was recently supported in a study by Alviggi *et al.*[7].

LH plays a key role in gonadal function by regulating the production of androgens, the precursor molecules of estrogens in *theca* cells. Although LH-receptors are expressed on human follicles already from the start of the cycle [8], the synergizing effect of LH and FSH appears to be most prominent from the mid-follicular phase and onwards. LH is a heterodimeric hormone characterized by two subunits, alfa and beta, produced in the anterior pituitary gland; the beta-subunit confers the specificity of the hormone. However, different types of LH with different biological and pharmacokinetic features have been identified. Thus, there are four genetic variant types of the beta-*subunit* of LH. The first type was discovered in 1992 when Weiss *et al.* reported a case of a young man affected by pubertal retardation [9]. The basal LH level in this patient was twice the normal level, however, obviously of a poor biological activity due to a substitution of a glycine to arginine in the 54^th^ position. During a study testing new monoclonal antibodies for LH measurement, another anomalous LH form was discovered [10]. The altered immune-reactivity was caused by a Trp^8^Arg mutation [11]. This variant (v-betaLH) seems to be significantly widespread in different ethnical groups (Table 1). Thus, the carrier frequency in Finland has been reported to be as high as 41.9%, while a significantly lower incidence was detected in Bengali inhabitants (about 2.56%). In Italy, the carrier frequency of this polymorphic variant is approximately 14%.

**Table 1:** The carrier frequencies of v-beta LH common variant in different ethnic groups

**Population**	**Sample ( *n *)**	**Frequency (%)**	**95% Confidence interval**
*Finland (Lapps)*	129	41.9	33.4 - 50.4
*Sweden*	376	18.9	14.9 - 22.9
*United Kingdom*	212	15.1	10.3- 19.9
*Italy*	294	13.9	9.7 - 26.4
*United States (black)*	251	14.7	10.3 - 19.1
*United States (hispanic)*	196	7.1	3.5 - 10.7

From Nilsson *et al.*[17], (modified).

Importantly, the v-betaLH is characterized by an extra glycosylation signal into the β subunit, which apparently adds a second oligosaccharide side-chain to Asn13 of the β protein. This molecular variation influences the pharmacokinetic properties of v-betaLH which shows an elevated bioactivity *in vitro*, but a significantly shorter half-life (26 min) in circulation when compared with the wild type LH (48 min) [12]. The aim of the present study was to corroborate previous findings indicating higher consumption of exogenous FSH in women with variant v-betaLH and to explore its frequency in a Danish female population.

## Methods

This is a retrospective analysis of the association between LH polymorphism and the outcome of COS and IVF. More specifically, samples examined in the present study were derived from a previously published trial [13], focusing on ovarian response in relation to mid-follicular serum LH levels. In that study, a total of 220 Danish women undergoing IVF or ICSI treatment in the Fertility Clinic at Skive regional hospital (Skive, Denmark) were prospectively enrolled in a consecutive manner. Exclusion criteria were: age > 40 years and baseline FSH >10 IU/L, any endocrine, genetic, systemic inflammatory-immunological disorder, polycystic ovarian syndrome, endometriosis and presence of one ovary, only. A frozen thawed serum sample from all patients was used to evaluate the presence of v-betaLH.

An Institutional Review Board approval was not required for the present study, as frozen serum samples derived from a previous study, approved by The Ethics Committee of Viborg County, during which each patient had given written consent [13]. Moreover, data was managed in a manner which excluded the identification of subjects.

### Ovarian stimulation

All patients underwent a long *gonadotropin-releasing hormone agonist* (GnRH-a) down-regulation protocol as previously described [13]. In brief, pituitary desensitization was induced by the administration of GnRH-a (Suprefact; Hoechst, Horsholm, Denmark) 0.8 mg s.c. daily from the mid-luteal phase for 12–20 days, followed by a reduction of GnRH-a to 0.4 mg s.c. daily. Ovarian stimulation was performed with r-hFSH (Gonal F; Serono Nordic, Copenhagen, Denmark or Puregon; MSD, Ballerup, Denmark), using an individualized dose between 100 IU and 375 IU s.c. daily according to antral follicle count, baseline FSH, *body mass index* (BMI) and age. The ovarian response was monitored by ultrasound examination starting on day 8 of stimulation and the dose of r-hFSH was adjusted if necessary. The ovulatory dose of *human chorionic gonadotropin* (hCG), 10.000 IU (Profasi; Serono Nordic, Copenhagen, Denmark) was administered when at least three follicles reached a mean diameter of 17 mm. Luteal phase support was given in the form of micronized progesterone (Cyclogest; Hoechst, Copenhagen, Denmark) twice daily or once daily (Crinone 8%; Serono Nordic, Copenhagen, Denmark), starting on the day following oocyte retrieval and continuing until the day of the pregnancy test (i.e. day 12 after embryo transfer). Oocytes were retrieved 35 h after the hCG injection.

### Blood samples and hormone assays

Blood sampling was performed: on stimulation day 1 (S1), day 8 (S8) on the day of triggering final oocyte maturation, and on the ovum pick-up (OPU) day. Aliquots were frozen at −20°C for subsequent analysis of estradiol, FSH, LH and androstendione. LH and FSH were measured by time-resolved immunofluorometric assay, the AutoDelfia spec.kit (Wallac Oy, Turku, Finland). Estradiol and androstenedione were measured according to manufacturers instructions using a commercially available RIA kit intended for measurements in serum (DSL-4200; Diagnostic System Laboratories, Texas, USA). All assays were intended for measurements in serum samples.

The hormone assays were carried out at the Fertility Clinic in Skive Regional Hospital (Skive, Denmark).

### V-betaLH Immunofluorometric assay

The venous blood (10 ml) was allowed to clot and centrifuged at 400 g for 10 min, where after serum was separated, divided into a maximum of four aliquots and frozen [13]. After thawing, the LH concentrations of all serum samples collected were determined by two different *immunofluorescent assay* (IFMA) as previously reported [14,15]. In brief, the total LH concentration was determined using LHspec (Wallac Oy, Turku, Finland) as this assay also recognizes v-betaLH [10]. The other assay, I3/A2, recognizes only the wild-type form (wt-LH) of LH. The I3/A2 assay was directly performed by one of the authors (K.P.) according to the protocol he previously developed and described [15]. A ratio of the two assays (wt-LH_I3/A2_ /total LH_LHspec_) was used to determine the LH status of these individuals. Previous studies have shown that heterozygotes for v-betaLH have a wt/total LH ratio between 0.2 and 0.9 as the I3/A2 assay recognizes only about half of the total immune-reactive LH in heterozygote serum. The ratio is >0.9 for wt-LH homozygotes and <0.2 for homozygous variants [16].

### Statistical analysis

The results are reported as the mean ± SD. Data were analyzed with the SPSS version 12.0 (SPSS Inc., USA). An ANOVA one-way model was used to compare continuous variables; χ^2^ statistics were used to compare discontinuous data. *Receiving operating characteristics* (ROC) curves were designed to find the best “cumulative dose” of r-hFSH, predictive of the highest probability to express V-betaLH variant. A *p* value <0.05 was considered statistically significant.

## Results

**Figure 1:**
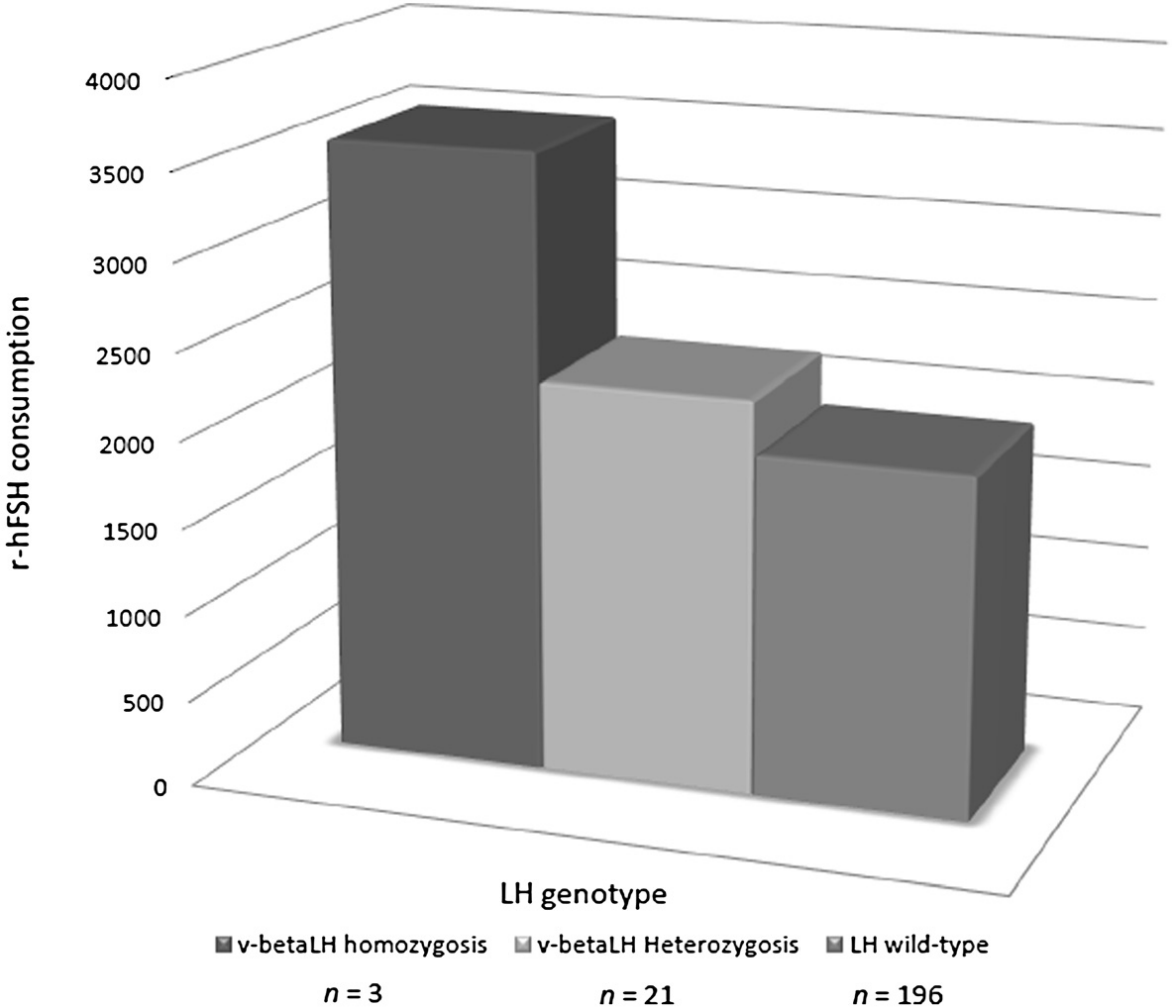
**Association between v-betaLH and different profiles of ovarian response to r-hFSH.** v-betaLH homozygosis (n = 3); v-betaLH heterozygosis (n = 21); LH wild type (n = 196). r-hFSH: recombinant human follicle-stimulating hormone; LH luteinizing hormone; v-betaLH: variant beta subunit luteinizing hormone.

A total of 220 IVF cycles were retrospectively evaluated. V-betaLH was observed in 10.9% of patients, with an allelic frequency of 12.2%. Patients were divided into two groups according to LH genotype. Group A consisted of 196 wt/wt women. Group B included 24 carriers of v-betaLH in (21 heterozygotic and 3 in homozygotic carriers). The two groups were comparable regarding age, BMI, cause of infertility and indication for IVF. Basal levels of FSH, LH and estradiol were also similar (Table 2). The outcome of the two groups is reported in Table 3. No statistically significant differences in estradiol and LH levels throughout COS and in the mean number of oocytes retrieved were observed between groups. Fertilization rates and pregnancy rates per cycle were also comparable. In contrast, the mean number of embryos transferred was significantly lower in group B compared to Group A (1.3 ± 0.6 versus1.6 ± 0.8, *p* = 0.042) (Table 3). Moreover, Group B received a marginal but statistically higher cumulative-dose of r-hFSH (1959.8 ± 736.45 *versus* 2435.86 ± 932.8 IU, *p* = 0.048) (Table 3). When one-way ANOVA in a within design was applied, the LH genotype (factor) had a statistically significant effect (*p* <0.01) on the cumulative dose of r-hFSH, which showed a progressive increase from wt/wt (1959.8 ± 736.45 IU) to v-betaLH hetero- (2267.5 ± 824.3) and homozygotic women (3558.3 ± 970.9) (Figure 1). Moreover, the androstenedione serum concentration on the day of triggering final oocyte maturation was statistically different between groups A and B (1.49 ± 0.76 *versus* 2.06 ± 1.28, *p =* 0.02). This difference disappeared on the day of oocyte retrieval (Table 3).

**Table 2:** Characteristics and infertility diagnosis of wild type and carriers of v-betaLH

	**Group A wild-type n = 196**	**Group B v-betaLH v n = 24**	**p-value A vs B**
Age (yrs)*	30.8 ± 4.0	30.5 ± 3.9	NS
*BMI (Kg/m*^*2*^)*	24.8 ± 3.1	25.3 ± 3.4	NS
*Basal E*_*2*_(nmol/L)*	0.4 ± 0.9	0.2 ± 0.15	NS
Basal FSH (UI/L)*	6.2 ± 1.8	6.7 ± 1.35	NS
Basal LH (UI/L)*	5.7 ± 2.7	5.4 ± 2.5	NS
***Infertility diagnosis***			
*Tubal factor (%)*	26.5	16.75	NS
*Male factor (%)*	32.7	29.15	NS
*Idiopathic form (%)*	16.3	16.75	NS
*Combined (%)*	16.3	29.15	NS
*Other (%)*	8.7	8.2	NS

*Values are reported as mean ± SD.

Abbreviation: *NS* not statistically significant.

**Table 3:** Ovarian response to stimulation and outcome of IVF treatment according to LH genotype

	**Group Awild-type LH n = 196**	**Group Bv-betaLH n = 24**	**p value A vs B**
*Estradiol Day S1 (nmol/L)*	0.1 ± 0.1	0.1 ± 0.1	NS
*Estradiol Day S8 (nmol/L)*	3.9 ± 2.8	3.4 ± 3.0	NS
*Androstenedione on Day of trigger (ng/mL)*	1.5 ± 0.8	2.1 ± 1.3	0.012
*Androstenedione on OPU (ng/mL)*	5.35 ± 2.4	5.53 ± 2.3	NS
*LH (IU/L) Day S1*	1.9 ± 0.9	2.1 ± 1.3	NS
*LH (IU/L) Day S8*	1.9 ± 1.25	1.8 ± 0.95	NS
*LH (IU/L) Day OPU*	0.1 ± 0.1	0.1 ± 0.7	NS
*Cumulative FSH dose (IU)*	1959.8 ± 736.45	2435.9 ± 932.8	0.048
N. mature oocytes retrieved*	8.6 ± 4.9	7.3 ± 4.85	NS
N. embryos transferred*	1.6 ± 0.8	1.3 ± 0.6	0.042
*Fertilization rate (%)*	52	46	NS
*Positive betahCG (%)*	37	45	NS

Values are expressed as mean ± SD.

Abbreviations: *NS* not statistically significant; *oPu* ovum pick up.

**Figure 2:**
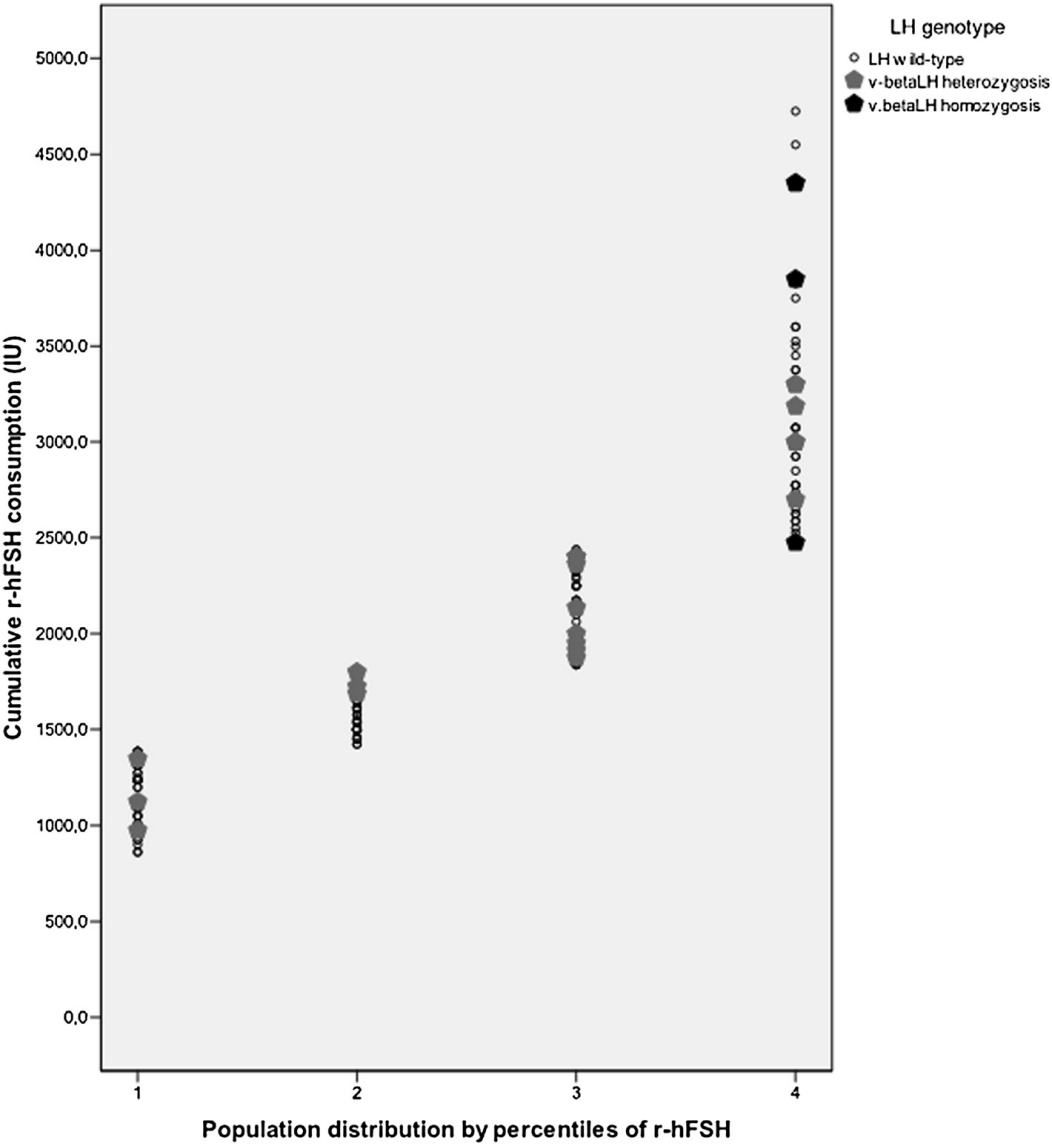
**Population stratified according by quartiles of cumulative r-hFSH dose.** 1^st^ quartile; 2^nd^ quartile; 3^rd^ quartile; 4^th^ quartile. r-hFSH: recombinant human follicle-stimulating hormone; LH luteinizing hormone; v-betaLH: variant beta subunit luteinizing hormone.

Population has been also stratified in quartiles group according r-hFSH consumption (Figure 2). The allelic frequency of v-betaLH was progressively 12.5%, 12.5%, 33.5% and 41.5% from the first to the fourth quartile.

**Figure 3:**
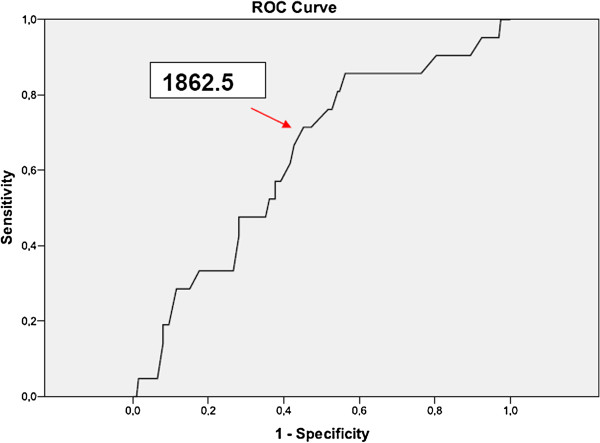
**ROC curve dependent variable cumulative dosage of FSH and independent v-betaLH expression.** Red dart shows the most predictive cut-off. Cumulative dosage r-hFSH (UI) *cut-off* 1862.5 IU. Sensitivity: 0.750; 1-Specificity: 0.444.

When a ROC curve was calculated (*Area under the curve value,* AUC 0.676), a cumulative dose of r-hFSH of 1862.5 IU was identified as the most predictive cut-off for the presence of the LH variant (*Sensitivity:* 0.75 – *1 -Specificity*: 0.44) (Figure 3). The study population was stratified into two subgroups on the basis of this cut-off value. Group 1 included 115 women who received a cumulative dose of r-hFSH ≤1862.5 IU; group 2 included 105 patients with a r-hFSH consumption >1862.5 IU. Considering groups 1 and 2, respectively, no statistically significant differences emerged in age (32.07 ± 3.85 *versus* 31.09 ± 3.74), BMI (24.01 ± 3.16 *versus* 23.9 ± 3.10 Kg/m^2^) and basal FSH (6.7 ± 1.71 *versus* 6.56 ± 1.83 IU/L) were similar between groups. When the LH genotype was evaluated, 6/115 (5.2%) and 18/105 (17.1%) carriers were found in the two groups, respectively (*p* <0.005).

## Discussion

This is the first study exploring the v-betaLH frequency in a Danish IVF population. V-betaLH was present in 10.9% of patients, whereas the allelic frequency was 12.2%, quite similar to that reported for a number of other European populations, however somewhat lower than that of the Finnish population [17].

The study confirms data reported in a previous paper [7], demonstrating that v-betaLH is associated with a higher consumption of r-hFSH during COS. More specifically, a relationship between the r-hFSH cumulative dose and the LH genotype was found, where the gonadotropin cumulative dose showed a positive trend in v-betaLH women. Although only three women with the variant in homozygosis were analyzed, it is interesting to remark that a sort of gradient in r-hFSH consumption was observed, with the lowest in the wt/wt group and the highest in v-betaLH homozygotic carriers (Figure 1). The mean number of oocytes retrieved was not related to the LH genotype (Table 3). This parameter was preferred to the number of dominant follicles as indicator of ovarian response. This choice was related to the fact that evaluation of dominant follicles, usually performed by different physicians, is more subjective, mainly when ovaries are “crowded”. In contrast, *ovum* pick up is a more standardized procedure which was performed by the same operator in all cases (P.H.). A cumulative dose of r-hFSH 1862.5 IU was identified as the most predictive cut-off for the presence of LH variant. Taken together, these data raise the question of whether v-betaLH assessment of all patients should be employed prior to ovarian stimulation, or whether one should await the results of the first stimulated cycle. Following this line, a good prognosis patient who, despite a starting dose of 150 IU of r-hFSH needed a cumulative dose higher than 2000 IU to achieve an apparently normal response (i.e. at least 5 oocytes retrieved) should be recommended for LH genotyping. If the patient was either homo-or heterozygotic for v-betaLH, the recommendation would be to add exogenous LH rather than increase the FSH dose during the subsequent cycle [2-4].

In the present study, serum samples from a previous study [13] were analyzed, which in turn made not possible the use of molecular biology approach (i.e., polymerase chain reaction). Nevertheless, the immuno-assay adopted is a well established method which has been validated in previous studies [16].

Wild-type and v-betaLH hormones are functionally different. More specifically, v-betaLH seems to possess a shorter half-life, however, with a more potent action at the receptor level when compared to the wild-type [12,18]. Thus, it is expected that the phenotypic expression of the hormone types may be different. Also the combination of the two forms of LH, i.e. heterozygous for wild-type and v-betaLH, could differ from both of the wt and v-betaLH homozygous phenotypes. Furthermore, a number of different LH-receptors genotypes exist and whether the interaction between the subtypes of LH and the different subtypes of the LH-receptor is unequal is currently unknown. Due to the low frequency of the v-betaLH homozygotes, no clear picture of the phenotypic effects of the variant has yet emerged. Several authors ascribe a higher activity to the variant LH due to elevated levels of serum estradiol, testosterone and sex hormone-binding globulin compared to wild-type LH women [19]. On the other hand, there is evidence that v-betaLH protects obese women from developing symptomatic polycystic ovarian syndrome [20]. Moreover, observational trials suggest that v-betaLH is related to a reduced gonadal function and subfertility [21]. Finally, differences in the circulatory kinetics between the two types of LH may explain the diversities in LH function between patients with ovulatory disorders and women with normal ovulatory cycles [22]. This study confirms that v-betaLH carriers need a higher FSH dose during COS, supporting the idea that this polymorphism represents a biologically less active form of LH, unable to adequately support FSH activity during follicular stimulation. In this series of patients no difference in terms of fertilization and pregnancy rates were found between groups, indicating that increasing r-hFSH dose during COS may counteract lower v-betaLH activity. Nevertheless, women with v-betaLH showed a statistically significant reduction in the mean number of embryos transferred (Table 3). This observation may be consistent with the evidence that cooperation between FSH and LH is also crucial in regulating finals steps of follicle-oocyte maturation, including resumption of oocyte meiosis [23-25]. Taken together, these aspects raise the question whether the use of higher doses of r-hFSH is able to totally overcome the impact of the LH variant on oocyte competence and on the outcome of IVF. This point could be addressed by further investigation in a larger study population. On the other hand, it could be argued that exogenous LH supplementation in v-betaLH carriers is capable of correcting ovarian response to FSH. This hypothesis is consistent with studies demonstrating that young, normogonadotrophic women showing slower and suboptimal response to exogenous r-hFSH benefit from r-hLH supplementation [2-4].

Interestingly, the serum androstenedione level on the day of triggering final oocyte maturation was significantly elevated in patients with v-betaLH. A previous study found that women following a standard long GnRH-agonist protocol stimulated with r-hFSH showed a significant positive association between serum levels of LH and androstenedione on day 8 of stimulation [26], supporting that LH has a pronounced effect on the circulatory concentration of androstenedione. This paradox may be related with the augmented FSH consumption in the v-betaLH group. In fact, FSH may *via* the granulosa cells increase the capacity of the theca cell compartment to produce androgens by stimulating the expression of *Cytochrome P450 17alpha-hydroxylase* (CYP17). This hypothesis is consistent with evidence from rats model [27].

## Conclusions

In conclusion, the present results provide, for the first time, information on the v-betaLH frequency in a Danish IVF population, confirming that carriers of the polymorphism require higher doses of r-hFSH during COS. The evidence supports the idea that v-betaLH represents a less bioactive variant of LH. Furthermore, on our way to an individualized-pharmacogenomic approach to COS, we suggest that LH genotyping provides important information for the clinician prior to ovarian stimulation.

On the bases of these results, it could be interesting to test the hypothesis that LH supplementation is able to improve both ovarian response and outcome of IVF in v-betaLH carriers. An adequate sample size should be calculated in a future RCT having v-betaLH carriers treated with only FSH as control group.

## Abbreviations

AUC: Area under the curve; BMI: Body mass index; COS: Controlled ovarian stimulation; CYP17: Cytochrome P450 17alpha-hydroxylase; FSH: Follicle-stimulating hormone; GnRH-a: Gonadotropin releasing hormone agonist; hCG: Human chorionic gonadotropin; ICSI: Intracytoplasmic sperm injection; IMFA: Immunofluorescent assay; IU: International unit; IVF: In vitro fertilization; LH: Luteinizing hormone; OPU: Ovum pick-up; ROC: Receiving operating characteristics; r-hFSH: Recombinant human follicle-stimulating hormone; r-hLH: Recombinant human luteinizing hormone; v-betaLH: Variant beta subunit luteinizing hormone; wt-LH: Wild type form luteinizing hormone.

## Competing interests

The authors declare that they have not competing interests.

## Authors’ contributions

CA, GD and PH: study design, data analysis and critical discussion. SL, AC, RC and AM: data analysis and critical discussion. PD and IS: critical discussion. KP: v-betaLH analyses and critical discussion. CYA: prospective data collection, data analysis and critical discussion. All authors read and approved the final manuscript.
